# The global, regional and national burden of peptic ulcer disease attributable to smoking from 1990 to 2021: A population-based study

**DOI:** 10.1016/j.pmedr.2025.103019

**Published:** 2025-02-22

**Authors:** Shuai Wang, Tao Zhang, Dongming Li, Xueyuan Cao

**Affiliations:** Department of Gastric and Colorectal Surgery, General Surgery Center, The First Hospital of Jilin University, Changchun, Jilin, China

**Keywords:** Peptic ulcer disease, Smoking, Global burden, Disability-adjusted life years, Mortality, Public health

## Abstract

**Background:**

Peptic ulcer disease (PUD) remains a significant global health challenge, with its prevalence generally declining due to advances in healthcare and reduction in key risk factors. However, smoking continues to be a major contributor to the burden of PUD. This study analyzes the temporal and spatial patterns of PUD burden attributable to smoking globally from 1990 to 2021, providing insights for public health interventions.

**Methods:**

Utilizing data from the Global Burden of Diseases, Injuries, and Risk Factors Study (GBD) 2021 database, we assessed deaths, disability-adjusted life years (DALYs), age-standardized mortality rate (ASMR), and age-standardized DALY rate (ASDR). Trends from 1990 to 2021 were evaluated using average annual percentage change (AAPC), and predictive analyses performed to understand past and future patterns.

**Results:**

In 2021, 29,390 deaths and 816,999 DALYs were caused by PUD attributable to smoking worldwide. From 1990 to 2021, deaths, DALYs, ASMR (AAPC: −4.05), and ASDR (AAPC: −4.18) showed significant declines globally. Males experienced a higher burden than females across all metrics. At the national and regional levels, low and low-middle socio-demographic index (SDI) areas exhibited higher ASMR and ASDR than high-SDI regions, with East Asia, South Asia, and Southeast Asia contributing the highest burden. Future projections indicate a continued decline in the burden of PUD attributable to smoking over the next decade.

**Conclusion:**

Despite global declines in the burden of PUD attributable to smoking, substantial disparities persist, particularly in underdeveloped regions. Focused anti-smoking policies and targeted resource allocation are necessary to reduce the disease burden and address regional inequalities.

## Highlights

• Peptic ulcer disease remains a significant global health challenge.
• Smoking is an important risk factor for peptic ulcer disease.
• Global burden of peptic ulcer disease attributable to smoking should not be ignored.

## 1 Introduction

Peptic ulcer disease (PUD) is a prevalent gastrointestinal disorder characterized by mucosal damage greater than three–five mm, extending into the submucosa (Sverdén et al., 2019). PUD primarily occurs in the proximal duodenum and stomach and is commonly classified as gastric or duodenal ulcers in clinical practice (Lanas and Chan, 2017; Liu et al., 2022). Irrespective of disease duration, PUD can lead to severe acute complications such as bleeding, perforation, or obstruction. Furthermore, chronic gastric ulcers increase the risk of stomach cancer (*Global Burden of 369 Diseases and Injuries in 204 Countries and Territories, 1990–2019: A Systematic Analysis for the Global Burden of Disease Study* 2019, 2020; Ren et al., 2022). These severe outcomes contribute to substantial medical and social burdens. The pathogenesis of PUD is multifactorial, with the primary causes being the use of non-steroidal anti-inflammatory drugs and infection with *Helicobacter pylori*. Historically, smoking was considered an insignificant risk factor for PUD and received limited attention (Yeomans, 2011; Eastwood, 1997). However, recent studies have increasingly highlighted smoking as a major risk factor for PUD (Liu et al., 2022; Yuan et al., 2023).

As of 2019, more than one billion people globally were current smokers, and this number is expected to rise in the coming decades. Since 1990, 113 out of 204 countries have experienced a notable increase in the number of smokers, with 111 countries showing a significant rise since 2005. The global tobacco epidemic has profound health and economic consequences, underscoring the urgent need for tobacco control as a key public health priority (*Spatial, Temporal, and Demographic Patterns in Prevalence of Smoking Tobacco Use and Attributable Disease Burden in 204 Countries and Territories, 1990–2019: A Systematic Analysis from the Global Burden of Disease Study 2019. Lancet (London, England*), 2021).

Two studies using the Global Burden of Diseases, Injuries, and Risk Factors Study (GBD) 2019 database have explored the global burden of PUD. These studies show that while the prevalence of PUD continues to rise, the global number of PUD-related deaths has decreased from 278,979 in 1990 to 236,139 in 2019, indicating a decline in mortality. However, the overall burden of PUD remains significant (Ren et al., 2022; Xie et al., 2022).

To date, no studies have assessed the global burden of PUD attributed specifically to smoking. This study aims to fill this gap by systematically evaluating global trends in the burden of PUD attributable to smoking, using data from GBD 2021. By integrating key indicators such as mortality and disability-adjusted life years (DALYs), this study provides insights into the global, regional, and country-level distribution and trends of PUD. These analyses are crucial for understanding the current burden of disease and will inform future public health strategies and research directions. By identifying high-risk populations and regions, more targeted interventions can be developed to effectively reduce the morbidity and mortality associated with PUD.

## 2 Methods

### 2.1 Data source

The data of this study come from the latest data set published by GBD 2021 (http://ghdx. healthdata.org/gbd-results-tool). GBD 2021 estimated the related indicators of 23 age groups from birth to 95 years old and above: male, female and the sum of all sexes. GBD 2021 classifies 204 countries and territories into 21 regions and seven super-regions based on geographic proximity and epidemiological similarities (Murray et al., 2012). Examples of GBD regions include East Asia, Eastern Europe, and Australasia. These 21 regions are further grouped into seven super-regions based on mortality patterns by cause: central Europe, eastern Europe, and central Asia; high income; Latin America and the Caribbean; north Africa and the Middle East; south Asia; southeast Asia, east Asia, Oceania; and sub-Saharan Africa (GBD 2021 Risk Factors Collaborators, 2024).

GBD 2021 encompasses 88 risks, including one aggregate of all risks, three Level-one risks, 20 Level-two risks, 42 Level-three risks, and 22 Level-four risks. Smoking is a level-three risk.

Smoking was defined as current daily or occasional use of any smoked tobacco product. PUD was defined as defects in the stomach (gastric ulcers) or the duodenum (duodenal ulcers) extending through to the muscularis mucosa into the submucosa.

Because the data of GBD 2021 are public, it didn't required ethical approval. The analytic process for this study complied with the Guidelines for Accurate and Transparent Health Estimates Reporting statements (***GATHER checklist***).

### 2.2 Statistical analysis

To analyze the data from 1990 to 2021, we used the following metrics: deaths, DALYs, mortality rate, DALY rate, age-standardized mortality rate (ASMR), age-standardized DALY rate (ASDR), and the population attributable fraction (PAF) with their 95 % uncertainty interval. Uncertainty interval was calculated based on 1000 draw-level estimates for each parameter. The 95 % uncertainty interval was defined as the 25th and 975th values across all 1000 draws. The PAF is defined as the percentage of a disease that will be eliminated if a certain risk factor is eliminated (GBD 2019 Diabetes and Air Pollution Collaborators, 2022). The mortality rate indicates deaths per 100,000 people, DALY rate represents DALYs per 100,000 people, ASMR reflects age-standardized deaths per 100,000, and ASDR denotes age-standardized DALYs per 100,000.

The GBD 2021 analysis uses a comparative risk assessment framework with seven interrelated steps. First, it quantifies the relative risk of health outcomes based on exposure to specific risk factors. Second, exposure data are collected, and exposure levels and distributions are estimated using Bayesian models (ST-GPR and DisMod-MR 2.1) to control for bias. Third, the theoretical minimum risk exposure level is determined based on epidemiological evidence. Fourth, PAFs are calculated to estimate health changes if exposure is reduced to theoretical minimum risk exposure levels. Fifth, the summary exposure value of each risk, representing age-specific risk-weighted exposure rates, is calculated. Sixth, mediation factors adjust PAF overestimation and account for interactions among risk factors. Finally, the attributable burden is calculated for each combination of age, sex, location, and year. (GBD 2021 Risk Factors Collaborators, 2024).

We used Joinpoint software to calculate the average annual percentage change (AAPC) to quantify the long-term trend of ASMR and ASDR from 1990 to 2021. The logarithmic age-standardized index can be applied to the regression model: ln (y) = α + βx + ε, where Y represents the corresponding age-standardized index, and X represents the calendar year. The calculation of AAPC is 100 × (exp (β)-1), and the model can also calculate its 95 % confidence interval (95 % CI) (Yang et al., 2021). Assume that the 95 % confidence interval of the corresponding AAPC estimate is greater than 0. In this case, the age-standardized index is considered to be rising. If the 95 % confidence interval is less than 0, it shows a downward trend, and if the 95 % confidence interval contains 0, it shows a stable trend. In addition, in order to explore the influence of the social demographic index (SDI) on the burden of PUD attributable to smoking, Spearman correlation analysis was used to evaluate the correlation at the national and regional levels, taking into account the non-normal distribution of the corresponding variables (Sang et al., 2022).SDI is a comprehensive measure of per capita lagging income distribution, average years of education and female fertility rate under 25 (GBD, 2021). It is closely related to health outcomes. The value range of SDI is 0–1, where 0 represents the lowest development level, and 1 represents the highest development level (Rezaei et al., 2023). In this study, 204 countries and regions are divided into five groups according to the SDI value: high SDI (> 0.805129), high-middle SDI (0.689504–0.805129), middle SDI (0.607679–0.689504), low-middle SDI (0.454743–0.607679) and low SDI (≤ 0.454743) (Yang et al., 2021).

Frontier analysis evaluates the performance and efficiency of decision-making units (such as countries or regions) in transforming inputs into outputs (Sultana et al., 2023). In this study, data envelopment analysis and stochastic frontier analysis are used to construct an efficiency frontier representing best practices, and the performance of each decision-making unit is compared with this frontier to determine the efficiency level. We have evaluated the performance of these countries and regions, and the lower limit of the ratio of ASMR to ASDR represents the lowest value that each country or region can achieve according to its SDI level.

The autoregressive comprehensive moving average (ARIMA) model consists of the autoregressive model and moving average model. Its basic assumption is that the data sequence is a random variable that changes with time. The ARIMA model can describe its autocorrelation and predict the future value according to the past value (Li et al., 2023). In this study, the ARIMA model was used to predict the ASMR and ASDR of PUD attributable to smoking in the next ten years.

Statistical analyses and data visualizations were conducted using R software (version 4.3.1) and Joinpoint software (version 5.2.0.0).

## 3 Results

### 3.1 Burden of PUD attributable to smoking in 2021

In 2021, the global number of deaths from PUD attributable to smoking was 29,390 (95 % uncertainty interval: 20,993, 39,938), with 3777 (95 % uncertainty interval: 2437, 5348) deaths in females and 25,613 (95 % uncertainty interval, 18,608, 34,760) in males (Table 1). The total DALYs for females were 94,063 (95 % uncertainty interval, 61,448, 131,010) and for males, 722,936 (95 % uncertainty interval, 520,869, 970,913), resulting in a global DALY count of 816,999 (95 % uncertainty interval, 582,192, 1,093,096) (***Table S1***).

**Table 1:** Global, regional, and sex-specific mortality burden of peptic ulcer disease attributable to smoking in 1990 and 2021, and its temporal trend.

Characteristics		1990			2021			1990–2021
		Deaths (95 %uncertainty interval)	ASMR/10^5 (95 %uncertainty interval)	Age-standardized PAF, % (95 %uncertainty interval)	Deaths (95 %uncertainty interval)	ASMR/10^5 (95 %uncertainty interval)	Age-standardized PAF, % (95 %uncertainty interval)	AAPC of ASMR (95 %confidence interval)
Overall		48,900 (34,568, 63,181)	1.23 (0.87, 1.59)	17.24 (12.3, 21.89)	29,390 (20,993, 39,938)	0.34 (0.25, 0.47)	12.48 (9.01, 15.96)	−4.05 (−4.15 - -3.95)
Sex								
	Male	42,812 (30,423, 55,667)	2.34 (1.65, 3.05)	24.84 (18.06, 31.31)	25,613 (18,608, 34,760)	0.65 (0.47, 0.88)	19.65 (14.11, 24.88)	−4.07 (−4.21 - -3.94)
	Female	6088 (4025, 8323)	0.3 (0.2, 0.41)	5.73 (3.86, 7.66)	3777 (2437, 5348)	0.08 (0.05, 0.12)	3.61 (2.4, 5.01)	−4.08 (−4.21 - -3.96)
5 SDI Regions								
	High SDI	6815 (4785, 8904)	0.62 (0.44, 0.81)	18.29 (12.94, 23.51)	3031 (2099, 4053)	0.15 (0.1, 0.2)	14.36 (9.91, 18.86)	−4.54 (−4.72, −4.36)
	High-middle SDI	9072 (6493, 11,646)	0.92 (0.65, 1.18)	21.74 (15.81, 27.33)	6580 (4738, 8666)	0.34 (0.24, 0.45)	17.38 (12.65, 21.83)	−3.12 (−3.46, −2.79)
	Middle SDI	14,615 (10,336, 19,380)	1.44 (1.01, 1.92)	18.4 (13.24, 23.37)	9342 (6556, 13,044)	0.36 (0.25, 0.51)	13.47 (9.64, 17.44)	−4.41 (−4.67, −4.15)
	Low-middle SDI	14,458 (9696, 20,200)	2.27 (1.51, 3.21)	15.98 (11.4, 20.58)	7870 (5227, 11,187)	0.56 (0.37, 0.8)	10.85 (7.72, 14.33)	−4.45 (−4.57, −4.32)
	Low SDI	3895 (2544, 5286)	1.67 (1.09, 2.26)	10.28 (7.16, 13.67)	2536 (1615, 3619)	0.5 (0.31, 0.7)	7.13 (4.94, 9.45)	−3.83 (−4.10, −3.57)
21 GBD Regions								
	Andean Latin America	145 (97, 206)	0.73 (0.48, 1.03)	9.19 (6.29, 12.41)	84 (53, 123)	0.14 (0.09, 0.21)	7.16 (4.9, 9.75)	−5.09 (−5.54, −4.64)
	Australasia	131 (89, 176)	0.56 (0.38, 0.75)	12.24 (8.28, 16.4)	29 (19, 42)	0.05 (0.04, 0.07)	8.73 (5.77, 12.02)	−7.33 (−7.93, −6.74)
	Caribbean	228 (157, 307)	0.88 (0.61, 1.19)	13.52 (9.44, 17.78)	169 (111, 236)	0.31 (0.21, 0.44)	10.46 (7.31, 13.89)	−3.22 (−3.80, −2.63)
	Central Asia	379 (272, 474)	0.76 (0.54, 0.95)	19.38 (14.1, 24.35)	453 (322, 608)	0.52 (0.37, 0.7)	15.82 (11.45, 20.35)	−1.15 (−1.91, −0.38)
	Central Europe	1562 (1134, 1976)	1.06 (0.76, 1.33)	21.5 (15.6, 27.09)	1163 (810, 1533)	0.57 (0.4, 0.75)	16.34 (11.51, 20.93)	−1.94 (−2.47, −1.40)
	Central Latin America	776 (557, 1006)	0.99 (0.71, 1.3)	11.54 (8.19, 14.98)	489 (328, 663)	0.2 (0.13, 0.27)	7.37 (5.12, 9.72)	−5.11 (−5.30, −4.93)
	Central Sub-Saharan Africa	160 (97, 256)	0.69 (0.41, 1.11)	6.8 (4.46, 9.51)	217 (122, 341)	0.36 (0.21, 0.58)	5.16 (3.13, 7.7)	−2.02 (−2.11, −1.93)
	East Asia	14,608 (10,109, 19,522)	1.78 (1.22, 2.37)	21.87 (15.78, 27.68)	8463 (5547, 12,603)	0.41 (0.27, 0.61)	18.99 (13.67, 24.32)	−4.67 (−5.02, −4.32)
	Eastern Europe	2197 (1655, 2723)	0.79 (0.6, 0.98)	23.98 (17.92, 29.78)	2591 (1889, 3313)	0.79 (0.58, 1)	18.6 (13.82, 23.34)	0.27 (−0.18, 0.72)
	Eastern Sub-Saharan Africa	885 (473, 1309)	1.05 (0.57, 1.54)	7.79 (5.42, 10.15)	927 (530, 1432)	0.46 (0.26, 0.7)	6.65 (4.53, 9.1)	−2.61 (−2.69, −2.53)
	High-income Asia Pacific	1169 (838, 1502)	0.61 (0.43, 0.79)	17.74 (12.81, 22.67)	446 (306, 603)	0.09 (0.06, 0.12)	13.26 (9.32, 17.34)	−5.97 (−6.21, −5.73)
	High-income North America	1244 (858, 1680)	0.35 (0.24, 0.47)	17.32 (11.86, 22.68)	608 (409, 829)	0.1 (0.06, 0.13)	14.11 (9.6, 18.55)	−4.08 (−4.28, −3.87)
	North Africa and Middle East	1323 (863, 1812)	0.81 (0.52, 1.13)	13.62 (9.31, 18.05)	899 (596, 1258)	0.21 (0.14, 0.3)	10.42 (7.06, 13.77)	−4.22 (−4.36, −4.08)
	Oceania	46 (30, 65)	1.5 (0.99, 2.11)	13.62 (9.63, 17.94)	57 (36, 86)	0.72 (0.46, 1.06)	11.88 (8.03, 15.76)	−2.37 (−2.53, −2.22)
	South Asia	14,730 (9817, 20,965)	2.47 (1.61, 3.52)	14.82 (10.23, 19.44)	7152 (4382, 10,467)	0.51 (0.31, 0.74)	9.65 (6.55, 12.88)	−4.97 (−5.17, −4.76)
	Southeast Asia	3808 (2475, 5518)	1.47 (0.95, 2.12)	19.89 (13.91, 25.83)	3017 (2103, 4222)	0.47 (0.33, 0.66)	13.38 (9.6, 17.28)	−3.59 (−3.67, −3.52)
	Southern Latin America	199 (143, 259)	0.43 (0.31, 0.56)	14.86 (10.58, 19.03)	104 (71, 139)	0.12 (0.08, 0.16)	11.84 (8.22, 15.57)	−3.80 (−4.59, −3.01)
	Southern Sub-Saharan Africa	228 (162, 303)	0.84 (0.59, 1.13)	15.28 (10.59, 20.44)	261 (178, 355)	0.44 (0.3, 0.59)	8.85 (6.11, 11.76)	−2.09 (−2.59, −1.59)
	Tropical Latin America	848 (614, 1082)	0.92 (0.66, 1.19)	21.08 (15.24, 27)	684 (467, 942)	0.27 (0.18, 0.37)	13.14 (8.88, 17.8)	−3.78 (−4.07, −3.50)
	Western Europe	3803 (2654, 4982)	0.65 (0.46, 0.85)	18.18 (12.71, 23.34)	1113 (751, 1516)	0.12 (0.08, 0.16)	13.57 (9.34, 17.85)	−5.41 (−5.51, −5.31)
	Western Sub-Saharan Africa	431 (286, 600)	0.46 (0.3, 0.66)	5.29 (3.55, 7.26)	465 (302, 646)	0.21 (0.14, 0.29)	3.7 (2.5, 5.01)	−2.46 (−2.55, −2.37)

ASMR, age-standardized mortality rate; PAF, population attributable fraction; AAPC, average annual percentage change; SDI, socio-demographic index.

**Fig. 1:**
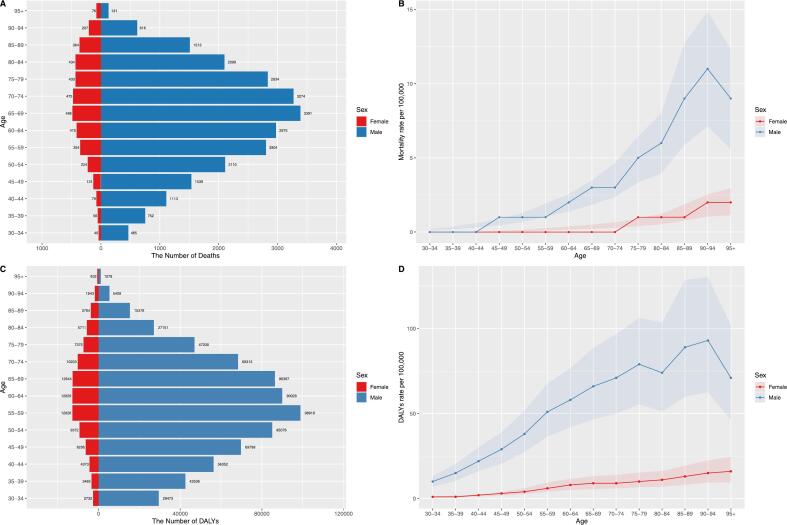
Age-specific number of deaths (A), mortality rate (B), number of disability-adjusted life years (C), and disability-adjusted life year rate (D) of peptic ulcer disease attributable to smoking in 2021 by sex. DALY, disability-adjusted life year.

The number of deaths, mortality, DALYs, and DALY rates were significantly higher in males than in females. For males, the peak number of deaths occurred in the 65–69 age group, the peak mortality and DALY rate were observed in the 90–94 age group, and the peak DALYs were in the 55–59 age group. In females, the peak number of deaths occurred in the 65–69 age group, the peak mortality and DALY rate were seen in those aged 95 and older, and the peak DALYs were observed in the 60–64 age group (Fig. 1).

At the SDI regional level, the middle SDI region recorded the highest number of deaths and DALYs, while the low-middle SDI region had the highest ASMR and ASDR. Among the 21 GBD regions, East Asia, South Asia, and Southeast Asia had the highest number of deaths and DALYs, whereas Eastern Europe, Oceania, and Central Europe had the highest ASMR and ASDR (Table 1 ***and Table S1***).

**Fig. 2:**
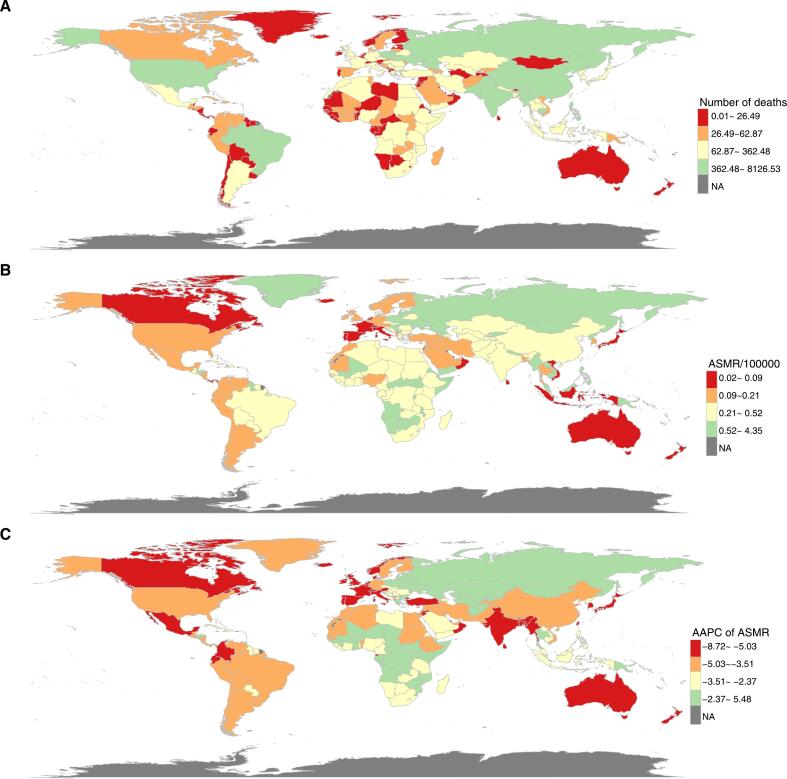
Global male and female mortality burden for peptic ulcer disease attributable to smoking. (A) Number of deaths in 2021; (B) Age-standardized mortality rate in 2021; (C) Average annual percentage change of age-standardized mortality rate from 1990 to 2021. ASMR, age-standardized mortality rate; AAPC, average annual percentage change.

At the country or territory level, China had the highest number of deaths and DALYs, followed by India and Russia (Fig. 2***, Fig. S1, Table S2***). Cambodia had the highest ASMR and ASDR, followed by Kiribati and Laos (Fig. 2***, Fig. S1, Table S2***).

### 3.2 Changing pattern of burden of PUD attributable to smoking from 1990 to 2021

**Fig. 3:**
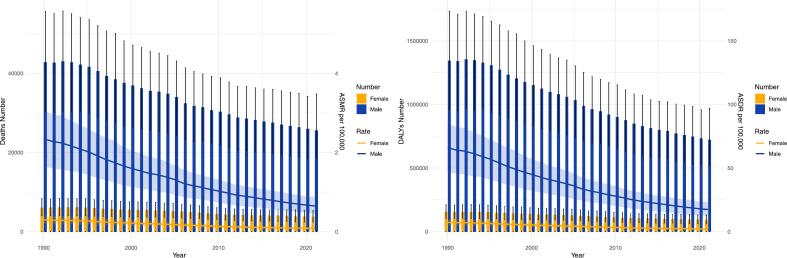
Trends in the number of deaths, the number of disability-adjusted life years, age-standardized mortality rate and age-standardized disability-adjusted life year rate of peptic ulcer disease attributable to smoking by sex from 1990 to 2021. (A) The number of deaths and age-standardized mortality rate; (B) The number of disability-adjusted life years and age-standardized disability-adjusted life year rate. DALY, disability-adjusted life year; ASMR, age-standardized mortality rate; ASDR, age-standardized disability-adjusted life year rate.

From 1990 to 2021, the global number of deaths from PUD attributable to smoking decreased from 48,900 (95 % uncertainty interval: 34,568, 63,181) to 29,390 (95 % uncertainty interval: 20,993, 39,938). The ASMR declined from 1.23 (95 % uncertainty interval: 0.87, 1.59) in 1990 to 0.34 (95 % uncertainty interval: 0.25, 0.47) in 2021, with an AAPC of −4.05 (95 % CI: −4.15, −3.95) (Table 1***,*** Fig. 3). DALYs and ASDR also decreased from 1990 to 2021, with an AAPC of ASDR of −4.18 (95 % CI: −4.27, −4.10) (***Table S1,*** Fig. 3).

At the SDI regional level, ASMR and ASDR declined in all regions. High SDI regions had the fastest decreases in ASMR, with AAPCs of −4.54 (95 % CI: −4.72, −4.36), while low-middle SDI regions experienced the fastest decreases in ASDR, with AAPCs of −4.81 (95 % CI: −4.92, −4.69) (Table 1 ***and Table S1***).

At the GBD regional level, ASMR and ASDR decreased in all regions except Eastern Europe. The fastest reductions were observed in Australasia and Asia, with AAPCs of −7.33 (95 % CI: −7.93, −6.74) for ASMR and − 6.86 (95 % CI: −7.48, −6.25) for ASDR (Table 1 ***and Table S1***).

At the country or territory level, Qatar saw the most significant reductions in both ASMR and ASDR (***Tables S2,*** Fig. 2***, Fig. S1***). South Korea and Ireland had the second and third fastest declines in ASMR, while the second and third fastest declines in ASDR were observed in South Korea and Ecuador.

### 3.3 PAF of the PUD attributable to smoking

The PAF for ASMR due to smoking varied by region. Globally, the PAF was 12.48 (95 % uncertainty interval: 9.01, 15.96), with the PAF for males (19.65 [95 % uncertainty interval: 14.11, 24.88]) significantly higher than that for females (3.61 [95 % uncertainty interval: 2.4, 5.01]) (Table 1).

At the SDI regional level, the highest PAF was observed in the high-middle SDI region (17.38 [95 % uncertainty interval: 12.65, 21.83]). At the GBD regional level, the highest PAF values were found in East Asia (18.99 [95 % uncertainty interval: 13.67, 24.32]), Eastern Europe (18.6 [95 % uncertainty interval: 13.82, 23.34]), and Central Europe (16.34 [95 % uncertainty interval: 11.51, 20.93]) (Table 1). In terms of age groups, the highest PAF was concentrated in individuals aged 40–65 years, with the peak at 55–59 years (18.54 [95 % uncertainty interval: 13.43, 23.66]) (***Table S3***). Similar patterns were observed for the PAF of DALY (***Tables S1 and S3***).

### 3.4 The changing patterns at different SDI levels

**Fig. 4:**
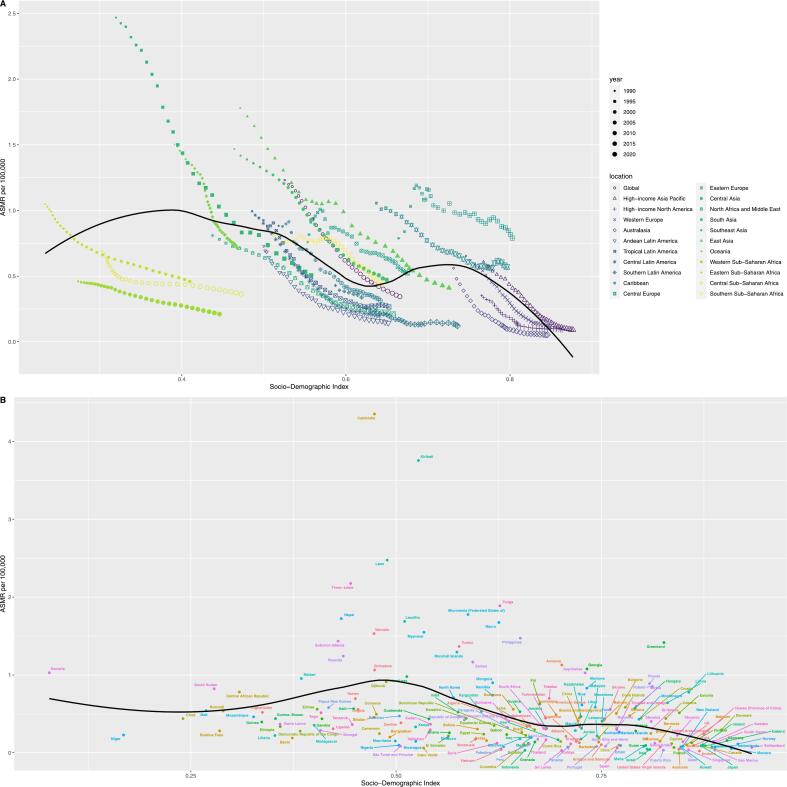
Distribution of age-standardized mortality rate for peptic ulcer disease attributable to smoking across different Socio-demographic Index levels. (A) Global and 21 Global Burden of Disease regions from 1990 to 2021; (B) 204 countries or territories in 2021. ASMR, age-standardized mortality rate; SDI, Socio-demographic Index.

At the level of 21 GBD regions, the relationship between ASMR and SDI was M-shaped, and ASMR gradually increased when SDI was less than 0.4. When SDI is about 0.4–0.6, ASMR gradually decreases. When SDI is about 0.6–0.7, ASMR gradually increases and then gradually decreases. The ASMR in low SDI and low-middle SDI regions was significantly higher than in middle, high-middle and high SDI areas. At the 204 national or territory levels, ASMR was highest in low-middle SDI countries. ASDR shows a similar trend (Fig. 4 ***and Fig. S2, Table S4***).

### 3.5 Frontier analysis of ASMR and ASDR

**Fig. 5:**
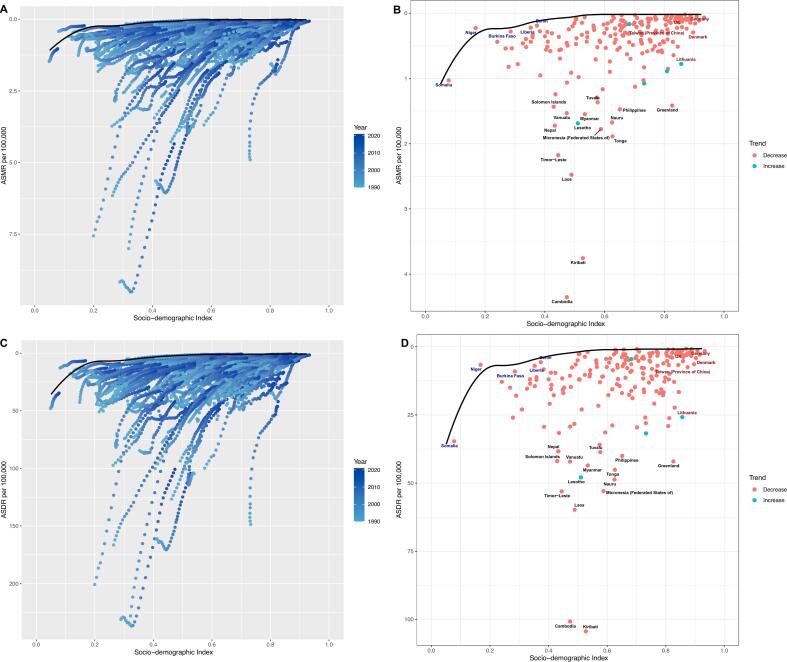
Results of frontier analysis for age-standardized mortality rate and age-standardized disability-adjusted life year rate of peptic ulcer disease attributable to smoking. (A) and (B): Frontier analysis for age-standardized mortality rate; (C) and (D): Frontier analysis for age-standardized disability-adjusted life year rate. ASMR, age-standardized mortality rate; ASDR, age-standardized disability-adjusted life year rate.

In the frontier analysis, the black line represents the lower limit of age standardization rate at different SDI levels, and the dots represent different countries or territories. The 15 countries or territories with the most significant effective differences in the world are marked in black font, the five countries or regions with the least effective differences in low SDI countries or territories are marked in blue font (Somalia, Niger, Burkina Faso, Liberia, Benin), and the five countries or territories with the most significant effective differences in high SDI countries or territories are marked in red font (Lithuania, Denmark, Taiwan (Province of China), UK, Germany). In Figs. B and D, the blue dots indicate the countries or territories where the age standardization rate decreased from 1990 to 2021, while the red dots indicate the countries or territories where the age standardization rate increased. With the improvement of SDI levels, the difference in disease burden among countries will decrease, and the less developed countries have great potential to reduce the burden. In contrast, the developed countries have low potential to reduce the burden. The analysis highlights 15 countries with the worst performance in the world, five countries with the best performance in low SDI regions, and five countries with the worst performance in high SDI regions. The disease burden is concentrated in countries or territories with an SDI of 0.4–0.6. Like Cambodia, Kiribati and Laos (Fig. 5).

### 3.6 Prediction of ASMR and ASDR of PUD attributable to smoking in the next decade

The ARIMA model predicted that the ASMR and ASDR for PUD attributable to smoking would continue to decline in both males and females. By 2030, the predicted values for males are 0.43 (ASMR) and 12.16 (ASDR), while for females, they are 0.06 (ASMR) and 1.49 (ASDR) (***Fig. S3 and Table S5***).

## 4 Discussion

This study systematically quantified the burden of PUD attributable to smoking and explored its temporal and spatial trends. From 1990 to 2021, the global impact of smoking on PUD gradually decreased, as evidenced by the reduction in the PAF, as well as a significant decline in deaths, DALYs, ASMR, and ASDR. The burden of PUD attributable to smoking was higher in males than in females. Overall, the burden of PUD attributable to smoking varies significantly by age, sex and regional differences.

Studies have shown that smoking contributes to PUD by constricting blood vessels in the mucosa, reducing its resistance and promoting ischemia (Andersen et al., 2000). Smokers also have higher levels of carboxyhemoglobin, and carbon monoxide exacerbates mucosal ischemia, facilitating ulcer formation (Smith et al., 1998). Smoking further impairs gastric and duodenal mucosal protection mechanisms and increases the risk of *Helicobacter pylori* infection, a known risk factor for PUD. Moreover, smoking can cause harmful duodenal contents to reflux into the stomach, exacerbating ulceration. Clinical studies have also shown that smokers are more likely to develop ulcers that are harder to treat (Parasher and Eastwood, 2000; Li et al., 2014). The majority of PUD-related deaths are caused by complications such as perforation or bleeding, which can be difficult to control (Sonnenberg, 2007). Smoking is a well-established risk factor for the perforation of gastroduodenal ulcers (Amalia et al., 2024; Svanes et al., 1997), reinforcing its role as a critical contributor to PUD morbidity and mortality.

Our findings align with previous studies indicating that the disease burden is substantially higher in males than females. This discrepancy may be partly due to the protective effects of female sex hormones, which enhance mucosal defense mechanisms, such as increasing mucus and phospholipid levels and bicarbonate secretion (Smith et al., 2008). Additionally, in 2019, the age-standardized smoking rate was 32.7 % for males and 6.62 % for females (*Spatial, Temporal, and Demographic Patterns in Prevalence of Smoking Tobacco Use and Attributable Disease Burden in 204 Countries and Territories, 1990–2019: A Systematic Analysis from the Global Burden of Disease Study 2019. Lancet (London, England*), 2021), contributing to the higher burden in males. Both sexes experience higher mortality and DALY rates in older age groups, indicating the need for increased attention to the elderly population.

Although the global ASMR and ASDR have declined over the past three decades, the disease burden remains considerable. In 2021, the global death toll was 29,390, and the DALY burden was 816,999. The highest numbers of deaths and DALYs were concentrated in China and India, which also rank first and second in the global number of smokers. Notably, 341 million (30 %) of the world's 1.14 billion smokers reside in China (*Spatial, Temporal, and Demographic Patterns in Prevalence of Smoking Tobacco Use and Attributable Disease Burden in 204 Countries and Territories, 1990–2019: A Systematic Analysis from the Global Burden of Disease Study 2019. Lancet (London, England*), 2021), contributing to the high disease burden in these countries. However, regions with low-middle and low SDI, such as Kiribati, Cambodia, and Laos, had higher ASMR and ASDR. These countries face challenges such as limited healthcare resources, low socio-economic status, and insufficient awareness of the risks associated with smoking. In contrast, high-SDI regions benefit from better medical infrastructure and healthcare systems (Xu et al., 2023), leading to lower ASMR and ASDR. Moreover, the per capita burden of PUD highlights the significant inequalities between regions: low-middle SDI regions with smaller populations bear a disproportionately high burden relative to their population size, further emphasizing the importance of targeted interventions in these areas.

East Asia, South Asia, and Southeast Asia were the top three regions for the number of deaths and DALYs, while Eastern Europe, Oceania, and Central Europe had the highest ASMR and ASDR. These disparities may be due to environmental and geographic factors, as well as differences in public health policies. To address these regional differences, countries should implement targeted tobacco control policies, such as increasing tobacco taxes, which have proven to be one of the most effective measures to reduce tobacco consumption (Taheri et al., 2024; Jha and Peto, 2014). As income levels rise, especially in developing countries, it is crucial to adjust tobacco tax policies to maintain their effectiveness (*Spatial, Temporal, and Demographic Patterns in Prevalence of Smoking Tobacco Use and Attributable Disease Burden in 204 Countries and Territories, 1990–2019: A Systematic Analysis from the Global Burden of Disease Study 2019. Lancet (London, England*), 2021). Our predictions using the ARIMA model suggest that ASMR and ASDR will continue to decline over the next decade, reflecting the positive impact of tobacco control efforts and improvements in healthcare systems. However, countries with high ASMR and ASDR need to prioritize interventions targeting smoking populations and encourage regular health screenings.

This study provides the first comprehensive analysis of the global, regional, and national burden of PUD attributable to smoking, utilizing the most recent epidemiological data from 204 countries or territories. It offers valuable insights into the trends and disparities in disease burden by geography, SDI, age, and sex. Notably, this study emphasizes the heavier disease burden in underdeveloped areas compared to developed ones.

Finally, we have to admit that this study has some limitations. Firstly, in GBD 2021, to estimate the mean exposure for each risk factor, systematic literature reviews were carried out. These reviews aimed to identify risk factor exposure studies published or discovered since GBD 2019. The data from these studies were then integrated with information from other sources, such as household and health examination surveys, censuses, ground - sensing or remote - sensing data, and administrative records.However, it's important to note that the estimation results are contingent upon the existing data sources. The accuracy of the GBD data is highly dependent on the quality and completeness of the data reported by different countries. This situation may give rise to potential errors, especially in regions where data reporting is limited or inconsistent. Secondly, GBD data do not encompass all populations or regions comprehensively; therefore, the findings primarily represent the general situation of specific areas rather than a truly global perspective. Thirdly, there is the potential for publication bias related to the relative risk estimates, which may influence the accuracy of the results. Fourthly, while this study focuses on smoking as a risk factor for PUD, it is important to acknowledge that other significant factors—such as *Helicobacter pylori* infection, alcohol consumption, psychological stress, and the use of non-steroidal anti-inflammatory drugs—also contribute to PUD development (Rosenstock et al., 2003; Levenstein et al., 2015). However, the GBD 2021 database only identifies smoking as a risk factor, limiting the scope of the analysis. Lastly, while the association between smoking and the burden of PUD is supported by established evidence, the study relies on population-level data and statistical modeling, which may not fully capture the complex interactions of risk factors in individual cases. Future research should consider integrating broader risk factors and more granular data to enhance the validity and applicability of the findings.

## 5 Conclusion

This study provides crucial data on the global burden of PUD attributable to smoking, highlighting its continued significance for public health worldwide. Although recent trends show a reduction in this burden, it remains a substantial challenge for global health systems. To further mitigate its impact, it is essential to develop targeted policies aimed at smoking control, tailored to the geographical distribution and epidemiological characteristics of PUD. Such efforts will be pivotal in reducing the overall disease burden and improving public health outcomes globally.

## Funding

This work was supported by grants from The Scientific and Technological Development Program of Jilin Province (No.20240402015GH, No.YDZJ202501ZYTS032).

## CRediT authorship contribution statement

**Shuai Wang:** Writing – review & editing, Writing – original draft, Validation, Methodology, Investigation, Formal analysis. **Tao Zhang:** Writing – review & editing, Writing – original draft, Validation, Methodology, Formal analysis. **Dongming Li:** Writing – original draft, Validation. **Xueyuan Cao:** Writing – review & editing, Writing – original draft, Supervision, Resources, Methodology, Investigation, Funding acquisition, Conceptualization.

## Declaration of competing interest

The authors declare that they have no known competing financial interests or personal relationships that could have appeared to influence the work reported in this paper.

## Data Availability

The data used in this paper were obtained from free database downloads and have been described explicitly in the text. Further inquiries can be directed to the corresponding author.

## Appendix A Supplementary data

Supplementary material 1 Image 1

Supplementary material 2 Image 2

Supplementary material 3 Image 3
