# Supplementary material for: The global, regional and national burden of peptic ulcer disease attributable to smoking from 1990 to 2021: A population-based study
Source: Prev Med Rep. 2025 Feb 22;51:103019. doi: 10.1016/j.pmedr.2025.103019 (PMC11908546; doi:10.1016/j.pmedr.2025.103019)
Supplement: Supplementary file 1 — Supplementary material 1 [file mmc1.docx]

***

***

***Figure S1.***

Global male and female disability-adjusted life years burden for peptic ulcer disease attributable to smoking. (A) Number of disability-adjusted life years in 2021. (B) age-standardized disability-adjusted life year rate in 2021. (C) average annual percentage change of age-standardized disability-adjusted life year rate from 1990 to 2021.

ASDR, age-standardized disability-adjusted life year rate; DALY, disability-adjusted life year; AAPC, average annual percentage change





***Figure S2.***

Distribution of age-standardized disability-adjusted life year rate for peptic ulcer disease attributable to smoking across different socio-demographic Index levels. (A) Global and 21 Global Burden of Disease regions from 1990 to 2021; (B) 204 countries and territories in 2021.

ASDR, age-standardized disability-adjusted life year rate.

***Figure S3.*** Predicted trends of age-standardized mortality rate and age-standardized disability-adjusted life year rate of peptic ulcer disease attributable to smoking over the next 10 years (2022–2030) by sex. Red lines represent the true trend of age-standardized mortality rate and age-standardized disability-adjusted life year rate of peptic ulcer disease during 1990–2021; yellow dot lines and shaded regions represent the predicted trend and its 95% CI. (A) age-standardized mortality rate for males; (B) age-standardized disability-adjusted life year rate for males; (C) age-standardized mortality rate for females; (D) age-standardized disability-adjusted life year rate for females.


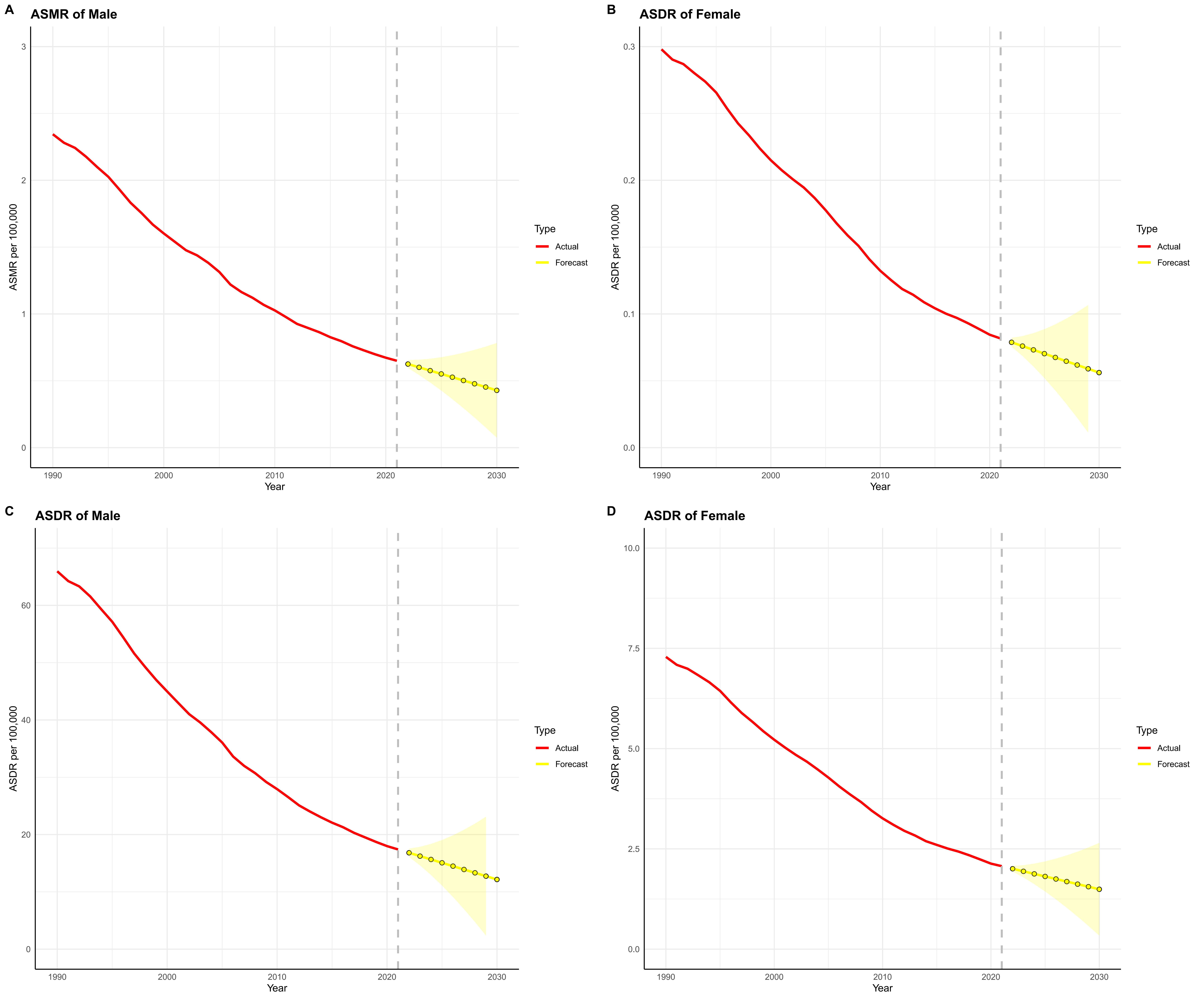


ASMR, age-standardized mortality rate; ASDR, age-standardized disability-adjusted life year rate
